# Supplementary material for: Intracardiac Echocardiogram: Feasibility, Efficacy, and Safety for Guidance of Transcatheter Multiple Atrial Septal Defects Closure
Source: J Clin Med. 2022 Apr 24;11(9):2394. doi: 10.3390/jcm11092394 (PMC9100238; doi:10.3390/jcm11092394)
Supplement: Supplementary file 1 [file jcm-11-02394-s001.zip › jcm-1660038-supplementary.pdf]

Supplementary Table S1. Basic characteristics and Outcomes of procedure using ICE for patients under 10kg of body weight

Variables	Total(%) N=185 (100)	B.Wt<10Kg(%) n=24(13)
**Age, year**		
**Mean ± SD**	22.1 ± 23.4	1.3± 0.5
**(range)**	(9 mo–77 y)	(9mo-1y 9mo)
**Bodyweight, kg**		
**Mean ± SD**	36.6 ± 25.1	8.7±1.2
**(range)**	(5–94.9)	(5-9.9)
**ASD defect count (%)**		
2	118 (63.8)	15(62.5)
3	39 (21.1)	4(16.7)
≥4	28 (15.1)	5(20.8)
**Distance between defects**		
**<7 mm**	120(64.9)	19(79.2)
**≥7 mm**	62(33.5)	4(16.7)
**Difficult to measure**	3(1.6)	1(4.2)
**Number of devices**		
**1**	146(79.3)	24(100)
**2**	33(17.9)	
**3**	5(2.7)	
**Procedural success rate**	182(98.4)	24(100)
**Immediate outcomes**		
**Complete closure**	8(4.4)	0
**Residual primary defects**	169(92.9)	11(45.8)
**Other isolated defects**	86(47.2)	13(54.2)
**Complication**	3(1.6)	0
**(device embolization,**		

**Complete AV block)**		
**Long term outcomes**		
**Complete closure**	116(63.7)	18(75.0)
**Residual primary defects**	35(13.2)	2(8.3)
**Other isolated defects**	42(23.1)	4(16.7)
**Complication & mortality**	0	0

B.Wt, body weight; SD, standard deviation; ASD, atrial septal defect
